# Occupational Exposure of On-Shift Ottawa Firefighters to Flame Retardants and Polycyclic Aromatic Hydrocarbons

**DOI:** 10.3390/toxics12090677

**Published:** 2024-09-17

**Authors:** William Papas, Rocio Aranda-Rodriguez, Xinghua Fan, Cariton Kubwabo, Janet S. L. Lee, Emma Fantin, Elita D. Zheng, Jennifer L. A. Keir, Dave Matschke, Jules M. Blais, Paul A. White

**Affiliations:** 1 Environmental Health Science and Research Bureau, Health Canada, Ottawa, ON, K1A0K9, Canada; william.papas@uwaterloo.ca (W.P.); xinghua.fan@hc-sc.gc.ca (X.F.); cariton.kubwabo@hc-sc.gc.ca (C.K.); jlee461@uottawa.ca (J.S.L.L.); emma.fantin@mail.mcgill.ca (E.F.); e8zheng@uwaterloo.ca (E.D.Z.); 2 Department of Biology, University of Ottawa. Ottawa, ON, K1N 6N5, Canada; jennifer.keir@hc-sc.gc.ca (J.L.A.K.); jules.blais@uottawa.ca (J.M.B.); 3 Ottawa Fire Services, Ottawa, ON, K1Z 7L9, Canada; dave.matschke@ottawa.ca

**Keywords:** firefighters, silicone wristbands, exposure assessment, passive sampler, PAHs, OPFRs, PBDEs

## Abstract

Firefighters can be exposed to complex mixtures of airborne substances, including hazardous substances released during structural fires. This study employed silicone wristbands (SWBs) as passive samplers to investigate potential exposure to polycyclic aromatic hydrocarbons (PAHs) and flame retardants (FRs). SWBs were deployed at different areas of four fire stations, in four truck cabins, and at an office control location; they were also donned outside the jackets of 18 firefighters who responded to fire calls. Overall, office areas had significantly lower PAHs than fire station areas. Vehicle bays and truck cabins had significantly higher concentrations of low molecular weight (LMW) PAHs than sleeping and living room areas. For organophosphate ester flame retardants (OPFRs), tri-n-butyl phosphate (TnBP) and tris(1-chloro-2-propyl) phosphate (TCPP) were detected in all the samples; 2-ethylhexyl diphenyl phosphate (EHDPP) was more frequently detected in the fire station areas. Triphenyl phosphate (TPP) concentrations were highest in the truck cabin and office areas, and tris(1,3-dichloro-2-propyl)phosphate (TDCPP) was highest in truck cabins. Thirteen of 16 PAHs and nine of 36 OPFRs were detected in all the SWBs worn by firefighters, and tris (2-butoxyethyl) phosphate (TBEP) was the predominant OPFR. Levels of LMW PAHs were significantly lower when firefighters did not enter the fire. LMW PAHs, HMW (high molecular weight) PAHs, and EHDPP were significantly elevated when heavy smoke was reported. This work highlights the potential for occupational exposure to PAHs and flame retardants in some fire station areas; moreover, factors that may influence exposure during fire suppression. Whilst firefighters’ occupational exposure to PAHs is likely related to fire suppression and exposure to contaminated gear and trucks, exposure to OPFRs may be more related to their presence in truck interiors and electronics.

## 1. Introduction

Firefighters work in conditions that are often associated with stress, dehydration, exhaustion, and excessive physical workload. They are also exposed to combustion emissions and heat. In addition, firefighters experience an elevated risk of injuries, chronic diseases, and certain types of cancer [1]. In a significant development, the International Agency for Research on Cancer (IARC) classified “occupational exposure as a firefighter” as a Group 1 carcinogen, i.e., a known human carcinogen [2]. This classification, supported by a growing body of evidence [1,3,4], underscores the importance of identifying the chemical contaminants firefighters are exposed to and understanding their correlations with increased incidence of certain cancers.

Fires, including municipal structural fires and wildfires, involve the combustion of a vast range of materials such as organic biomass (e.g., wood), electronics, construction materials, furnishings, textiles, and consumer products. Combustion of these materials produces and/or releases complex and hazardous emissions containing gases (e.g., carbon monoxide, nitrogen dioxide, and hydrogen cyanide), volatile organic compounds (VOCs) (e.g., benzene, styrene, and formaldehyde), dust and combustion-derived particulate matter (e.g., PM10), fibers (e.g., asbestos), metals (e.g., Sb, Pb, Cd, As), and semi-volatile organic compounds (SVOCs) (e.g., flame retardants, per- and polyfluoroalkyl substances (PFAS), polychlorinated biphenyls (PCBs), dioxins, furans, and polycyclic aromatic hydrocarbons (PAHs)). For detailed information regarding the chemical composition of combustion emissions and the toxicological properties of substances in combustion emissions, the reader is referred to IARC Monograph 132 [2].

Flame retardants (FRs) are compounds added to consumer products and building materials to comply with flammability standards. One important group of FRs is the polybrominated diphenyl ethers (PBDEs), which are widely used in building materials and consumer products. Concerns relating to their environmental persistence, bioaccumulation, and toxicity resulted in the prohibition of marketing or use of Penta- and OctaBDE by the European Union in 2003 [5]. Penta and OctaBDE were later phased out in the USA in 2005, followed by Canada in 2008. DecaBDE was phased out in the USA in 2013 [6]. As alternatives to PBDEs, organophosphate ester flame retardants (OPFRs) are incorporated into electronic products, plastics, textiles, and furniture to reduce their flammability [6]. In addition, OPFRs are increasingly utilized as plasticizers or in personal care products such as nail polish and hair sprays. Exposure to OPFRs has been linked to genotoxic and endocrine system effects, developmental effects, and certain cancers [7,8]. 

PAHs formed during the incomplete combustion of wood, oil, and other organic materials made of hydrocarbons are widely recognized for the occupational risk posed to firefighters [9]. Exposure to PAHs, historically monitored through urine sampling, personal air monitoring, dermal wipes, and passive sampling devices, has been linked to various health issues, including skin irritation, vomiting, kidney damage, and various cancers [2]. The carcinogenic hazard of PAHs is of particular concern since several have been evaluated by IARC and categorized as possible (Group 2b), probable (Group 2a), or known (Group 1) human carcinogens [10]. 

Despite wearing extensive personal protective equipment (PPE) (i.e., turnout gear) when responding to active fire events, firefighters can be exposed to VOCs and SVOCs through dermal contact with debris or contaminated PPE, inhalation, or accidental non-dietary ingestion [11,12]. Consequently, firefighters who have recently attended fires have been shown to have elevated levels of urinary metabolites of PAHs [13,14] and OPFRs [15] and elevated PBDE blood levels [16,17].

As previously noted, a complex mixture of substances is released during an active fire, and turnout gear used during active fire suppression can become contaminated, constituting a secondary exposure source [18]. Used and soiled turnout gear can contain high levels of PAHs [11] and other chemicals that can off-gas to areas in the fire truck cabins and fire stations [12,13]. Although some fire station designs allow for the isolation of living quarters from more contaminated areas, such as turnout gear storage areas and vehicle bays, this may not be the case for older stations and/or stations located in remote or small communities. Over time, these compounds can accumulate in fire stations, as evidenced by detectable levels of PAHs and PBDEs in dust within fire stations and truck cabins [12,19,20]. 

Passive sampling is a widely used technique for monitoring trace levels of VOCs and SVOCs in air. Unlike other exposure monitoring techniques, such as biomarker monitoring (e.g., metabolites in urine) or active air sampling, passive sampling methods offer study design and deployment duration flexibility; they are non-invasive and do not require maintenance during deployment. Moreover, passive samplers deployed with on-shift firefighters can easily be taken to fire suppression events. Silicone wristbands (SWBs) are particularly useful and convenient passive sampling devices. Since their introduction by O’Connell et al. (2014) to monitor PAH exposure of roofers [21], SWBs have been employed to study PAH exposures in natural gas workers [22] and inhabitants of coal mining communities [23]. Several studies involved the placement of SWBs on the wrist of firefighters attending a fire suppression event [24,25,26]. These studies focused on the impact of fuel type on airborne contamination level [27] and worker protection factors associated with various types of PPE [28,29]. 

Although many studies have investigated firefighter exposures during fire suppression, more data are needed to identify the risks posed by other sources of contamination, e.g., sources within fire stations. This work employed SWBs to investigate the potential for firefighters to be occupationally exposed to PAHs, PBDEs, and OPFRs in truck cabins and various locations within the fire station. In addition, SWBs were worn by firefighters involved in emergency fire suppression. The latter portion of the study complements our earlier work that evaluated the utility of SWBs for monitoring firefighter exposure [29].

## 2. Materials and Methods

### 2.1. Fire Station Sample Collection

Samples were collected between November 2021 and March 2022. Four fire stations (FS1–FS4) and one administrative office of the Ottawa Fire Services (OFS) participated in the study. All sampling locations were located in Ottawa, Canada. FS1 and FS4 are located in suburban areas, whereas FS2 and FS3 are in urban residential areas. FS2 is located next to a busy urban street, and FS3 is near a highway (i.e., <300 m distance). The OFS administrative office area is adjacent to a fire station, linked by a corridor with doors at each end. This was a large area composed of different offices and cubicles and provided a control to compare with fire station SWB levels.

Three SWBs were used to collect airborne contaminants in each of the selected rooms in the fire stations. They were hung from pre-cleaned metal hooks for 30 days in the following areas of the fire stations: vehicle bay (VB), sleeping quarters (SP), living room area (LR), and turnout (bunker) gear storage room (BS). These areas were chosen as they represent different potential exposure points for firefighters. All the LRs were areas adjacent to an open-concept kitchen where gas stove burners were used. The SP areas were located away from the VB and BS. BS rooms were usually adjacent to the VB and physically separated by doors. BS and VB were also separated by doors and corridors from the LR and SP. Only one fire station had a self-contained breathing apparatus (SCBA) decontamination room (DC) in which three SWBs were also deployed. In addition, three SWBs were deployed in one truck cabin (TR) in each fire station. Two trucks were manufactured in 2013 (FS1–TR and FS4–TR) and two in 2021 (FS2–TR and FS3–TR). A total of 10 SWBs were deployed throughout the office location. Field blanks were also collected, i.e., SWBs transported to each fire station and office area but not deployed. After completion of each deployment, SWBs were placed in their original plastic bags and stored at −20 °C until analysis.

### 2.2. Firefighter Sample Collection

The study protocol was approved by the Health Canada-Public Health Agency of the Canada Research Ethics Board (i.e., REB # 2021-009H approved 9 July 2021). Fifty-two participants were recruited from different OFS stations throughout the city of Ottawa. Informed consent forms were completed by all participants. In addition, an intake survey was conducted to collect demographic and occupational information for each participant, including metrics such as age, years in the fire service, and response team type. The firefighter sampling period extended over approximately six months. During this time, firefighter participants kept two SWBs in sealed bags in a turnout gear pocket until they were called to an active fire event. Once a call was received, firefighters attached two SWBs to the outside of their jackets prior to responding to the incident. Upon returning to the fire station, firefighters placed the SWBs into their original bags and stored them in a freezer at the fire station until transportation to the lab, where they were stored in a −20 °C freezer until analysis. In addition, participants completed a post-fire questionnaire that included questions regarding the type and duration of the event, and the role of the individual in the response. 

### 2.3. Chemicals and Standards

The list of target chemicals and their abbreviations are provided in Table S1. A 16 PAH native standard mix (EPA-PAH-STK), 14 PAH labeled internal standard mix (L429-IS), 9 PBDE native standard mix (BDE-MXF), and7 PBDE labeled internal standard mix (MBDE-MXFS) were purchased from Wellington Laboratories (Guelph, ON, Canada). OPFR standards were purchased individually from several suppliers, including AccuStandard (New Haven, CT, USA), CDN Isotopes Inc. (Pointe-Claire, QC, Canada), and Wellington Laboratories (Guelph, ON, Canada) (Table S1). Terphenyl d-14 recovery standard was purchased from Cambridge Isotope Laboratories (Andover, MA, USA). OmniSolv grade n-hexane, ethyl acetate, isopropyl alcohol, and pentane were purchased from Sigma-Aldrich (Oakville, ON, Canada). Optima-grade methanol and acetone were purchased from Fisher Scientific (Ottawa ON, Canada).

### 2.4. Materials

Silicone wristbands were obtained from two different sources. SWBs purchased from 24HourWristbands (Houston, TX, USA) were used for QA/QC purposes (i.e., method blanks and spike recoveries). They were cleaned using seven sonication cycles of 60 min each (see below). The first three cycles consisted of an ethyl acetate:hexane mixture (1:1), followed by one cycle of a mixture of ethyl acetate:methanol (1:1), and finally, three cycles of pentane. The SWBs were dried under a gentle stream of nitrogen and stored in a vacuum oven at 200 °C until analysis. Pre-cleaned SWBs purchased from MyExposome (Corvallis, OR, USA) were deployed to firefighters and fire stations using their original bags. 

### 2.5. Sample Preparation

Silicone wristbands were extracted using a sonication and soaking method adapted from Romanak et al. (2019) [30]. The samples were removed from their bags and cut in half using acetone-rinsed scissors. One half (~2.29 g) was thoroughly rinsed using deionized water to remove any surface particles, rapidly rinsed with isopropyl alcohol to remove residual water, and placed in a 60 mL glass vial. Then, an isotopically labeled internal standard mix was spiked directly onto the SWBs. The samples then underwent two extraction cycles; the first sonication step was performed with 35 mL of a mixture of hexane:acetone (7:3) for 2 h, and the vial was left on the benchtop to soak overnight. The solution was then transferred to a clean vial. The SWBs underwent a second extraction using 35 mL of hexane:acetone (1:1) for 2 h. The extracts from both sonication cycles were pooled together, concentrated to 0.70 mL using a Biotage Turbovap II (San Jose, CA, USA), and spiked with recovery standard for analysis. 

### 2.6. Analysis

The analytical instrument used for PAH and PBDE analysis was a Thermo Fisher Scientific TSQ 8000 Gas Chromatograph-triple quadrupole mass spectrometer (GC-MS/MS) (Cambridge, MA, USA). A Trace GC Ultra gas chromatograph fitted with a Phenomenex Zebron ZB-Semivolatiles column (Torrance, CA, USA) was used to achieve the separation. The GC injector was split/splitless injector operated in the splitless mode. The injection volume was 2 µL, and the carrier gas was helium at a constant flow rate of 1.0 mL/min. Analytes were eluted to the TSQ-8000 mass spectrometer operated in a multiple reaction monitoring (MRM) mode (Table S2). For OPFR analysis, samples were analyzed using an Agilent 7890N gas chromatograph (Santa Clara, CA, USA) coupled with a Waters Xevo TQ-GC mass spectrometer (Milford, MA, USA). The GC column was a Phenomenex Zebron ZB-5MS (Torrance, CA, USA). The carrier gas was helium with a constant flow of 1 mL/min. The GC injector was equipped with a programmable temperature vaporizer inlet (PTV) operated in solvent vent mode. The MS was operated in MRM mode (Table S2). Two MRM transitions were monitored for each target compound, one for quantitation and the other for confirmation. Comprehensive instrument method details are provided in the Supplementary Information (SI).

### 2.7. Quality Assurance/Quality Control

For each batch of samples, a procedure blank, method blank, and method spike were included to verify the integrity of the analysis. Field blanks (MyExposome SWBs) were included in each fire station and office area (n = 5). Results are shown in Table S3.

### 2.8. Statistical Analysis

For statistical analyses, samples were divided into two groups. The primary exposure source group comprised SWBs from firefighters responding to active fire events. The secondary exposure source group comprised SWBs deployed to the fire stations and fire trucks. The samples collected at the office building were considered control samples. SWB concentration values for specific compounds were omitted from the analyses and sum determinations if less than 20% of samples were above the method detection limit (MDL) (Table S3). If more than 80% of the samples were above the MDL, non-detect values were replaced with the MDL value divided by the square root of 2. If 20% to 80% of the samples were above the MDL, non-detect values were replaced with values calculated using NDExpo Version 1.0 (http://expostats.ca/site/app-local/NDExpo/, accessed on 11 November 2022). NDExpo imputes values using robust regression on order statistics [31]. The resulting SWB concentration data were analyzed using desktop SAS v9.4 (SAS Institute, Cary, NC, USA). The relationship between SWB concentrations (ng/g) and SWB deployment location was examined using analysis of variance (ANOVA). Similarly, relationships between SWB concentrations and fire attributes were examined using ANOVA. Where necessary, the data were log-transformed to equalize the variance across the range of observations, and post-hoc comparison of mean values employed Duncan’s multiple range test (MRT). Statistical significance was defined as *p* < 0.05. PAHs were divided into two groups based on the number of rings: low molecular weight (LMW) PAHs included those with fewer than 4 rings (i.e., naphthalene, acenaphthene, acenaphthylene, fluorene, phenanthrene and anthracene). High molecular weight (HMW) PAHs included those with 4–6 rings (i.e., pyrene, fluoranthene, benz[*a*]anthracene, chrysene, benzo[*b*]fluoranthene, benzo[*k*]fluoranthene, benzo[*a*]pyrene, indeno[*1,2,3-cd*]pyrene, dibenz[*a,h*]anthracene, and benzo[*g,h,i*]perylene).

## 3. Results

### 3.1. Fire Stations

#### 3.1.1. Polycyclic Aromatic Hydrocarbons (PAHs)

**Figure 1:**
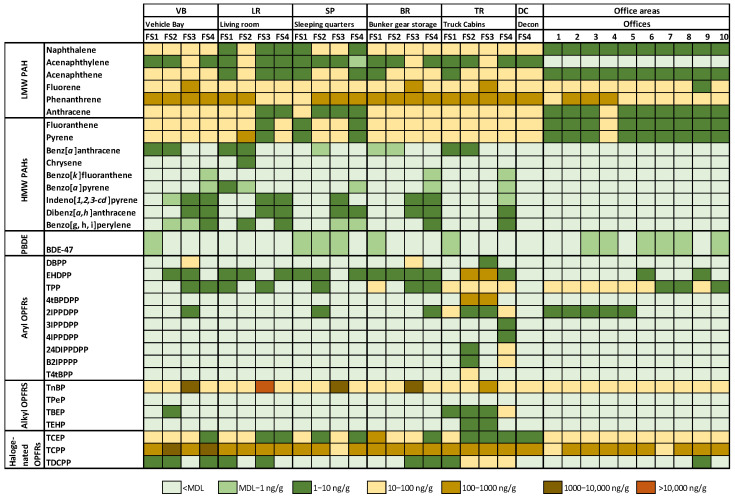
Heat map showing the geometric mean (GM, n = 3) concentrations of each targeted analyte in different areas (i.e., VB, LR, SP, BS, TR, DC, and offices) in each fire station (FS1–FS4).

The detection frequencies for each targeted compound in each area in the fire stations and offices (n = 10) examined are depicted in Figure S1. It is important to note that analytes with a detection frequency below 20% were omitted from the statistical analysis; however, they are included in Figure S1 (e.g., chrysene and some OPFRs) and Figure 1. The presence of naphthalene, acenaphthene, fluorene, phenanthrene, anthracene, fluoranthene, and pyrene in all the samples, including the office areas, indicates the widespread distribution of these PAHs. Acenaphthylene was detected in all the samples collected at the fire stations but not in the office areas. HMW PAHs had lower detection frequencies in the different areas of the fire stations, with higher detection frequencies in the VB. Surprisingly, HMW PAHs were detected in half of the samples collected in the LRs.

**Figure 2:**
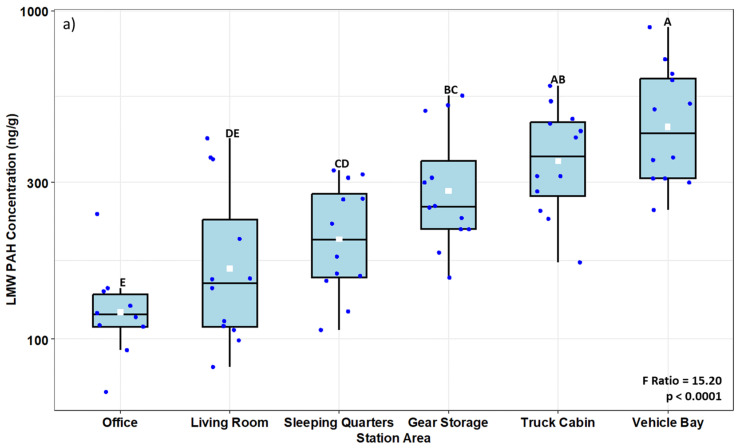

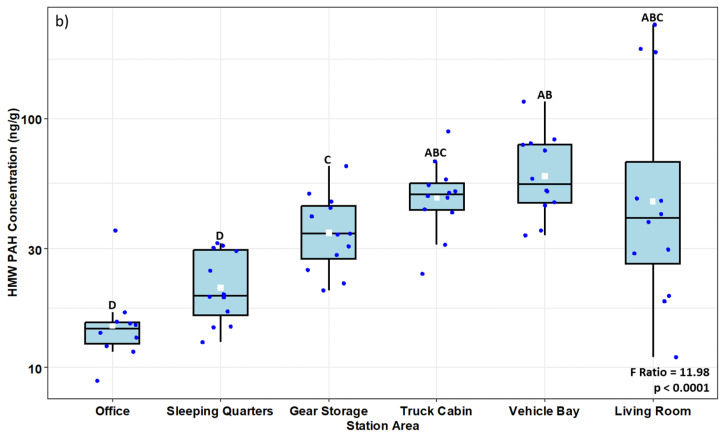
Box plots illustrating concentrations (ng/g) of (**a**) low molecular weight PAHs (LMW PAH) and (**b**) high molecular weight PAHs (HMW PAH) extracted from the SWBs deployed in different areas of the fire stations and office areas. Box limits represent the interquartile range (i.e., 25th to 75th percentile), the white squares represent the geometric mean values, the blue dots indicate the values for each observation, the solid line represents the group median, and the whiskers extend to the 5th and 95th percentiles. Boxes with different letters are significantly different at *p* < 0.05. The results of ANOVAs for sampling area effects are shown on the bottom right.

A heat map of the geometric mean (GM) concentrations per room (n = 3) in each fire station (FS1–4) is shown in Figure 1. With respect to the PAH levels, phenanthrene was the predominant LMW PAH in all the samples at concentrations between 2- to 10-fold higher than the other LMW PAHs (55 ng/g to 404 ng/g). Phenanthrene concentration was higher in the VB (GM 259 ng/g) when compared to other areas in the fire stations (109 ng/g to 191 ng/g) and the office area (94 ng/g) (*p* < 0.05). Fluoranthene and pyrene were the predominant HMW PAHs; the concentrations were approximately two-fold higher than the other HMW PAHs. Fluoranthene was consistently highest in VB and TR samples and significantly lower in the office area (*p* < 0.05). Moreover, there were significant differences between rooms in the fire stations. For example, VB and TR had significantly higher LMW PAH levels (GMs of 442 ng/g and 348 ng/g, respectively) compared to the other areas in the fire stations (Figure 2a). SP and LR areas had the lowest LMW PAH concentrations (GM 200 ng/g and 164 ng/g, respectively). In contrast, HMW PAHs were significantly higher in VB and TR and, surprisingly, in LR as well (GM 59 ng/g, 48 ng/g, and 47 ng/g, respectively) compared to BS and SP. Office areas and SP had the lowest concentration of HMW PAHs (Figure 2b). Overall, the HMW PAHs, LMW PAHs, and total PAHs were significantly lower in the office areas than in the fire station areas. There were no significant differences in LMW PAH and HMW PAH concentration values between VB and TR.

The data analyses also investigated station area differences in PAHs that have been categorized by the U.S. Environmental Protection Agency (USEPA) as “probably carcinogenic to humans” (i.e., B2 Carcinogens BaP, BaA, BbF, BkF, Chry, DBahA, Inde) [32]. The results indicate that there are no significant differences between the station areas examined for Inde, Diben, or BaA. The lack of an effect for these individual PAHs is likely related to the fact that they were infrequently detected (Figure S1). With respect to the level of total B2 PAHs, the results indicate that SWB levels are low (GMs of 1.6 to 2.6 ng/g) relative to either LMW or HMW PAHs (GM ranges of 121–442 ng/g and 15–59 ng/g, respectively). Interestingly, the results indicate a marginally significant effect of station area (*p* = 0.045) on total B2 PAHs, with levels in LR and VB (GMs of 2.6 ng/g and 2.4 ng/g, respectively) being significantly elevated relative to SP (GM of 1.6 ng/g). The lack of a more considerable effect of station area on total B2 PAHs, including previously noted elevations in both VB and TR, is likely influenced by the fact that the other four B2 PAHs were rarely detected (i.e., BaP, Chry, BbF, BkF, Figure S1).

To examine relationships among the PAHs investigated, the data analyses also included a Pearson correlation matrix of the SWB concentration values (Table S5). The results indicate strong to moderate correlations (i.e., 0.4 to 0.6 [33]) between the various LMW PAHs investigated. This is not surprising given the similarities in physical-chemical properties and the expectation that the investigated PAHs are components of complex, combustion-derived mixtures [34,35]. There were relatively few strong correlations among the HMW PAHs, most of which are infrequently detected (e.g., Chry, BkF, BbF, BaP, Figure S1).

#### 3.1.2. Flame Retardants

Figure 1 also includes an illustrative overview of the geometric mean (GM) concentrations of FRs per room (n = 3) in each fire station (FS1–4). Among the nine PBDEs included in the analysis, only BDE-47 was detected in the SWB samples. BDE-47 had a higher detection frequency in sleeping quarters (50%) and office areas (60%) and it was not detected in any of the LR samples (Figure S1). There was no significant difference in BDE-47 concentrations between the two rooms (Figure S2a).

**Figure 3:**
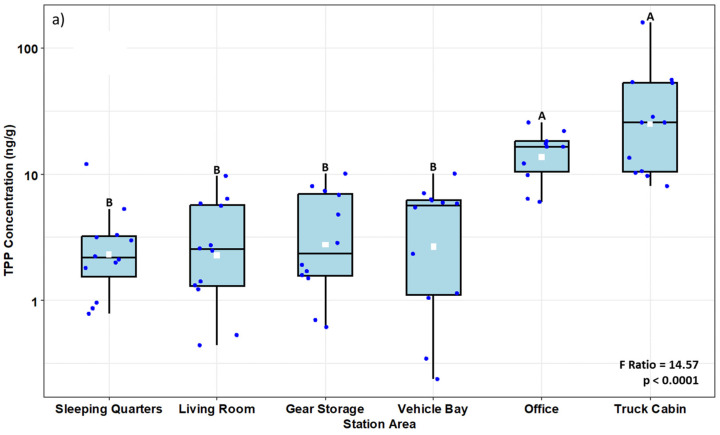

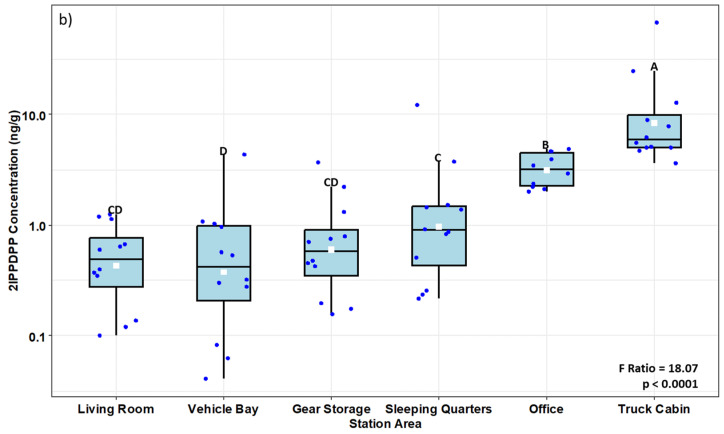
Box plots illustrating concentration (ng/g) of (**a**) TPP and (**b**) 2IPPDPP in the fire station areas. Box limits represent the interquartile range (i.e., 25th to 75th percentile), the white squares represent the geometric mean values, the blue dots indicate the values for each observation, the solid line represents the group median, and the whiskers extend to the 5th and 95th percentiles. Boxes with different letters are significantly different at *p* < 0.05. The results of ANOVAs for sampling area effects are shown on the bottom right.

Of the 36 OPFRs included in the analysis, only 17 were detected, at least in one sample. OPFR results unveiled interesting detection frequency patterns: tri-n-butyl phosphate (TnBP) and tris(1-chloro-2-propyl) phosphate (TCPP) were found in all the samples, including the office areas (Figure S1). The third most frequently detected OPFR was tris(2-chloroethyl)phosphate (TCEP), which was detected in over 92% of the samples. Some OPFRs were unique to the fire stations; for instance, 2-ethylhexyl diphenyl phosphate (EHDPP) was found in over 50% of the fire station samples but only in 20% of the office area samples. Certain OPFRs (i.e., tris(2-ethylhexyl) phosphate (TEHP), 3-Isopropylphenyl diphenyl phosphate (3IPPDPP), 4-Isopropylphenyl diphenyl phosphate (4IPPDPP), 2,4-diisopropylphenyl diphenyl phosphate (24DIPPDP), bis(4-isopropylphenyl) phenyl phosphate (B4tBPPP)) were exclusively found in samples collected above the back seats in the truck cabins. Finally, triphenyl phosphate (TPP) was detected in all the samples from the office area and TR. Unlike PAHs, there was significant variability in some of the OPFR samples between fire stations. For example, samples collected in fire station #3 (FS3) contained a markedly higher concentration of TnBP than the other three stations. In contrast, TCPP concentration was higher in the VB of fire stations #2 and #4. TPP concentrations in the TR and office area were significantly higher than the rest of the samples collected in the fire station (Figure 3a). 2IPPDPP levels were highest in the TR, followed by office area and SP. However, there were no differences in the levels found in the SP, BS, VB and LR (Figure 3b). Tris(1,3-dichloro-2-propyl)phosphate (TDCPP) levels were higher in the TR compared to the fire station (Figure S2b). Lastly, tris(1-chloro-2propyl)phosphate (TCPP) levels were higher in TR, VB, and BS compared to other station areas; there were no differences between LR, SP, and office areas (Figure S2c).

To examine relationships among the FRs investigated, the data analyses also included a Pearson correlation matrix of the SWB concentration values (Table S5). The results indicate some moderate correlations (e.g., 0.4 to 0.6, [33]) between several FR compounds. For example, there are noteworthy correlations between TPP and EHDPP (r = 0.67, *p* < 0.001), as well as between TDCPP and both TPP and EHDPP (r = 0.73 and r = 0.68, respectively). This likely results from their use as flame retardants and plasticizers. There was a weak correlation between TPP and 2IPPDPP (r = 0.28). Lastly, there are some moderate correlations between LMW PAHs and OPFRs such as EHDPP, TPP, and TDCPP. This may be a consequence of similarities in physical–chemical properties that govern SWB sorption.

### 3.2. Firefighter Exposure

#### 3.2.1. PAHs

A total of 52 participants enrolled in the study. Of those, only 24 firefighters were involved in fire suppression. Of those 24, only 18 firefighters attached the SWB to their jackets and returned them. In total, 18 SWBs were deployed to fire suppression events (Table S4). Only six firefighters (FF) reported the SWBs exposure duration (i.e., 20 to 90 min). Most firefighters responded to a residential fire, with the exception of one responding to a commercial fire, one to an industrial fire, and one to an automotive fire. Six firefighters did not enter the structure, and one reported attending a fire at the incipient stage (i.e., shortly after ignition). Others responded to fires that were growing (2 FFs) or in decay (5 FFs).

Thirteen PAHs were detected in the SWBs. Notably, Inde and DbahA were only detected in 3 SWBs and 1 SWB, respectively. The predominant PAHs quantified in the SWBs were naphthalene and acenaphthylene, accounting for more than 78% of the total PAHs. In SWBs with lower PAH concentration (i.e., <30 ng/g), naphthalene and acenaphthylene contributed less than 70% to the total PAHs. The concentration of PAHs in the 18 SWBs worn during on-shift fire suppression showed significant variability, with LMW PAHs concentration ranging between 9 ng/g and 7390 ng/g.

**Figure 4:**
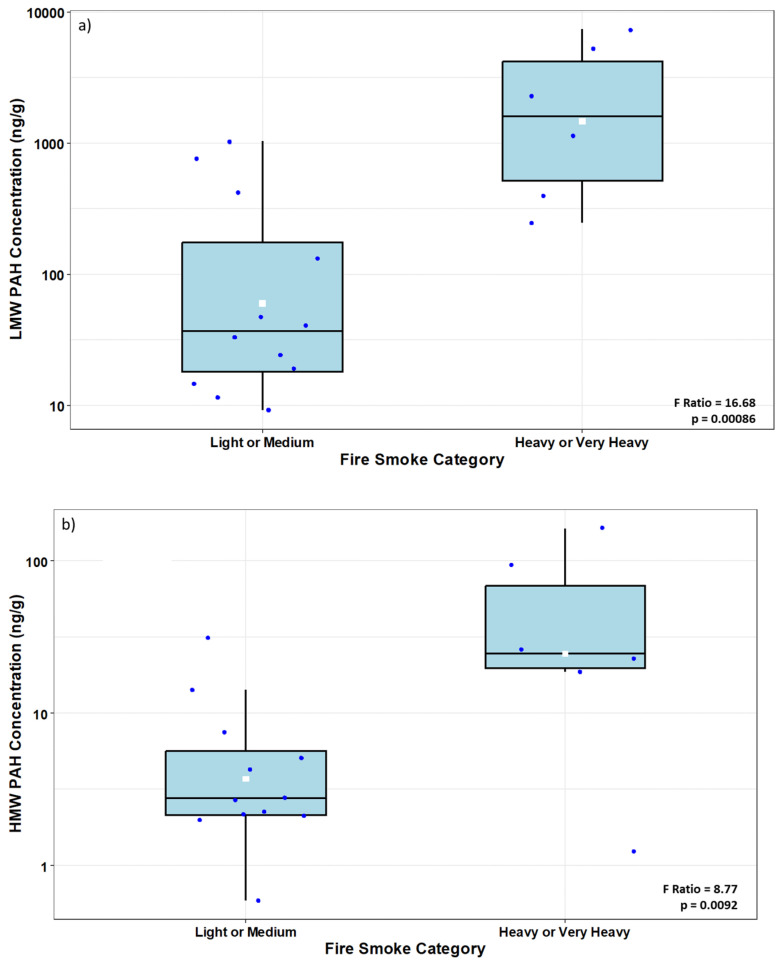
Box plots summarizing concentration (ng/g) of (**a**) low molecular weight PAH (LMW PAH) and (**b**) high molecular weight PAH (HMW PAH) in silicone wristbands donned outside the jacket of firefighters responding to a fire. Box limits represent the interquartile range (i.e., 25th to 75th percentile), the white squares represent the geometric mean values, the blue dots indicate the values for each observation, the solid line represents the group median, and the whiskers extend to the 5th and 95th percentiles. The results of ANOVAs for smoke level effects are shown on the bottom right.

Firefighters’ activities and smoke characteristics were significantly related to SWB PAH concentrations. LMW PAHs were significantly lower among firefighters who did not enter the fire (GM = 27 ng/g) compared to those who entered the fire (GM = 569 ng/g) (*p* < 0.002). In addition, HMW PAHs were significantly lower among firefighters who did not enter the fire (GM = 3 ng/g) compared to those who entered the fire (GM = 13 ng/g) (*p* < 0.03). Interestingly, the SWB level of B2 PAHs was not affected by reported firefighter activity. Records regarding the observed smoke level were also correlated with the concentration of PAHs found in the SWBs. For example, higher concentrations of LMW PAHs and HMW PAHs were found in samples collected when heavy or very heavy smoke was reported (GM = 1463 ng/g and 25 ng/g, respectively) compared to when light or medium smoke was reported (GM = 60 ng/g and 4 ng/g, respectively) (Figure 4a,b). Interestingly, the SWB level of B2 PAHs was not significantly affected by the reported smoke level (i.e., at *p* < 0.05); however, the effect was marginally significant at *p* = 0.051. Heavy or very heavy smoke was associated with higher levels of B2 PAHs (GM = 7 ng/g) relative to that associated with light or medium smoke (GM = 1.7 ng/g).

Similar to the fire station results, a correlation matrix was used to examine relationships between the concentrations of the various PAHs. As indicated in Table S6, there are strong correlations between the concentrations of both LMW and HMW PAHs. This is not surprising given the aforementioned similarities in physical–chemical properties and the expectation that the investigated PAHs will be present in complex combustion-derived mixtures [35,36].

#### 3.2.2. Flame Retardants

Out of the nine PBDEs analyzed, only BDE-47, BDE-99, and BDE-153 were detected in one SWB (1.1 ng/g, 0.6 ng/g, and 0.5 ng/g, respectively). For OPFRs, nine were detected in all the samples (i.e., EHDPP, TPP, 2IPPDPP, B2IPPP, TnBP, TBEP, TEHP, TCPP, and TDCPP). TBEP had the highest concentration with a GM of 43 ng/g (concentration range of 2.7 to 863 ng/g). The two OPFRs with the highest maximum concentration were TCPP (between 0.1 to 300 ng/g, GM = 4.7 ng/g) and EHDPP (between 0.1 to 340 ng/g). However, B4tBPPP and TPP had the highest GM concentrations (10 ng/g and 8.4 ng/g, respectively). Unlike PAHs, the concentration range and GM of individual OPFRs were generally not attributed to whether the firefighter entered the fire or not, nor the reported intensity of the smoke. Only EHDPP concentration was elevated during heavy to very heavy smoke, compared to light to medium smoke (*p* < 0.05) (Figure S3).

The correlation matrix summarized in Table S6 was also used to examine the relationships between various FRs investigated. The results indicate strong correlations between TPP, TnBP, and 2IPPDPP (r = 0.88 and 0.98, respectively), as well as correlations between both TBEP and TDCPP (r = 0.58) and several of the measured PAHs. Interestingly, there were some moderate correlations between some LMW PAHs and OPFRs, such as EHDPP, TPP, and TDCPP.

## 4. Discussion

### 4.1. Fire Stations

In this study, SWBs were used as a cost-effective sampling strategy to measure potential exposures to a comprehensive range of PAHs. The approach constitutes a low-burden method to study participants belonging to a high-exposure occupation. We examined areas in the fire stations that may be contaminated with PAHs and flame retardants. LMW PAHs were detected in all the SWB samples collected in the fire stations and office areas, with the exception of acenaphthylene. Several studies have shown that SWBs are effective at collecting LMW PAHs and, to a certain extent, some HMW PAHs. The latter was observed in SWBs that were worn on the wrist of FFs involved in fire suppression events [36]. The results obtained in the present study showed similar trends as those reported in studies that used other sampling methods [37,38,39]. For example, indoor air samples from the living quarters of 15 fire stations in Australia were collected using polyurethane foam-glass fiber filter (PUF-GFF) air samplers [19]. The dominant PAHs were phenanthrene, fluorene, pyrene, and anthracene, with median concentrations of 9.9, 2.7, and 1.8 ng/m^3^, respectively. In the present study, the same PAHs had the highest concentrations, corroborating the concentration trends. However, SWBs effectively collected 2–3 ring PAHs such as naphthalene, acenaphthylene, and acenaphthene, which were not reported by Banks et al. (2020) [19].

In terms of PAH distribution in fire stations, a study conducted in Poland [39] showed that PAH concentrations were higher in the changing room of two fire stations (5050–5925 ng/m^3^) followed by the vehicle bay (2110–3650 ng/m^3^) and common area (1880–2710 ng/m^3^). In another study, air samples were collected in vehicle bays of three fire stations, in addition to office areas and one truck cabin [11]. Total PAH air concentrations in the vehicle bays were significantly higher than in the office areas (i.e., 12-fold). Furthermore, total PAHs inside the truck cabin were significantly higher than the VB (i.e., 26-fold). The present study corroborates these findings as the GM concentrations inside the truck cabins and vehicle bays were higher than in the office areas, albeit there were no differences in LMW PAH and HMW PAH concentration values between VB and TR. In the present study, measurements of total PAHs were lower in office areas compared to other areas in the fire station. This suggests that the source of PAHs is either contaminated turnout gear or fire trucks entering the fire station. It also demonstrates that the separations between rooms were effective at reducing the cross-contamination between the fire station areas. Although this study showed that HMW PAHs in the LR were comparable to the levels found in VB and TR, this may be due to the high variability in the measurements. The source of HMW PAHs in the LR is hitherto unknown. Regarding the carcinogenic risk posed by the B2 PAHs detected in the various station areas, risk assessment was not possible since this study did not assess actual occupant exposures. At any rate, although the levels of HMW PAHs were elevated in VB, TR, and LR, 4 of the 7 PAHs denoted as B2 (i.e., BaP, Chry, BbF, BkF) were rarely detected. Indeed, BbF, a noteworthy B2 PAH, was never detected. Further studies might employ biomonitoring methods (e.g., urinary concentrations of PAH metabolites) to quantify PAH exposures associated with the occupation of the station during a specified shift when an emergency call was not obtained.

With respect to PBDEs, only BDE-47 was detected in the sleeping quarters and living room areas. The presence of BDE-47 in both areas was expected since, despite the current global restrictions on PBDE use, they were widely used in consumer products such as foam, mattresses, carpets, and furniture [15]. Its detection is likely related to the age of the furniture used in these areas.

The OPFRs findings in this study are significant. In the aforementioned Australian study [19], TnBP, TCEP, TCPP, TDCPP, TBEP, TPP, EHDPP, and TEHP were detected in all air samples collected in the living room areas of the fire stations, and TCPP had the highest concentration. In the present study, TnBP, TCPP, and TCEP were detected in more than 92% of the samples, with TCPP having the highest concentration. The ability to sample more areas provided a map of OPFR distribution in different areas of fire stations, as well as the truck cabins. These findings are particularly relevant to firefighter occupational exposure and can direct future exposure studies. For example, TPP levels were higher in the office area and truck cabins, and TCPP was higher in BS, TR, and VB compared to the office area. While some OPFRs (e.g., TPP) may be ubiquitous in occupational settings (including offices), others may be uniquely relevant to the firefighter environment. This variability could be associated with the use of Firemaster 550 (FM550), which is common in household furniture and contains TPP and isopropylated triaryl phosphates (ITPs) such as 2IPPDPP [40]. FM-550 and FM-600 are mainly added in flexible polyurethane (PU) foam, which is widely used in furniture such as sofas and seat cushions. Nevertheless, a weak correlation (r = 0.28, *p* < 0.05) was found between TPP and 2IPPDPP (Table S5). On the other hand, strong positive correlations were found between TDCPP and EHDPP (r = 0.68, *p* < 0.001) and TPP and 2IPPDPP (r = 0.73, *p* < 0.001), which could be attributed to their use as both flame retardants and plasticizers [41].

SWBs were also deployed inside the truck cabins. In another study using SWB as passive air samplers to examine the interior of different vehicles during the summer and winter [40], TEP, TIPB, TnBP, and TDCPP were found at higher detection frequencies than the rest of the OPFRs included in the study. The OPFR with the highest concentration was TCPP, with median concentrations of 62.9 and 231 ng/g in winter and summer, respectively. In the present study, the median concentration of TCPP was 217 ng/g for a sampling period of 30 days. Furthermore, more OPFRs were detected in the truck cabins (i.e., EHDPP, TPP, 2IPPDP, TnBP, TBEP). This is a unique contribution, as the more extended sampling period allowed the detection of OPFRs with more prolonged linear uptake (i.e., more than 60 days). Studies have shown that OPFRs take months to reach equilibrium between the air and SWB concentrations [42]; consequently, more OPFRs can be detected with longer sampling periods. The presence of OPFRs in the truck cabins and their detection pattern can likely be attributed to the use of flame retardants to comply with the Federal Motor Vehicle Safety Standard (FMVSS) no. 302 [43], which specifies the burn rate of the interior materials used in motor vehicles.

The results reported herein contribute to a more comprehensive understanding of the distribution of OPFRs in the firefighter occupational environment, and the extended sampling period provided more details regarding OPFRs released to the environment. These results underscore the importance of considering long-term exposure in occupational health and safety assessments. However, it is essential to note that our study was limited to a specific geographic area and time period. Therefore, findings may not be generalizable to all fire stations and truck cabins. For instance, the distribution of PAHs in fire station areas may differ from those reported in this study, particularly if the living quarters are not isolated from more contaminated areas such as turnout gear storage areas and vehicle bays. Furthermore, variations in decontamination protocols will influence the transport of chemicals to the fire station. Relatedly, variations in the types of fires encountered in a given area (e.g., wildland, structural, vehicle, etc.) will likely affect the nature of the combustion emissions encountered and the types of substances transported to the stations. In the case of flame retardants, their distribution in fire stations and truck cabins could be highly dependent on the flammability standards set by different jurisdictions.

### 4.2. Firefighter Exposure

The air quality during on-shift fire suppression was investigated using SWBs donned outside the jackets of firefighters who attended a fire suppression scene. PAHs with 2–5 rings were detected in all the samples, though concentrations in each SWB varied significantly (i.e., by up to three orders of magnitude). Although several B2 PAHs were detected in the SWB samples examined, the levels were comparatively low, and 4 of the 7 compounds were rarely detected. Interestingly, a recent study by Keir et al. (2023) followed the same approach of donning SWBs outside the jacket to examine firefighter training scenarios; the authors reported GM values that are one order of magnitude higher than the present study (i.e., 1040 ng/g for naphthalene versus 125 ng/g in this study) [29]. In the present study, six firefighters reported that they did not enter the fire, whereas all the firefighters participating in the Keir et al. study entered the live training fire inside a shipping container. In another study [28], which was conducted to assess protective factors associated with the use of different PPE configurations, SWBs were suspended in a shipping container used to mimic the conditions of a residential fire. The mean concentration of LMW PAHs in that study, which deployed SWBs for less than 10 min, was two orders of magnitude higher than that observed herein, where SWBs were deployed for a longer time period (i.e., 20–90 min). It is possible that differences in fuel and temperature and lack of movement of the suspended SWBs contributed to such differences.

In other studies that employed silicone-based passive samplers (i.e., bands or dog tags) to assess firefighter exposures [24,25,44], the turnout jacket protected the samplers. Nevertheless, all the studies reported higher LMW PAH detection frequencies and median concentrations when participants attended fires compared to those who did not.

Few firefighter studies included other chemicals in the analysis of silicone-based passive samplers. Poutasse et al. (2020) conducted a screening analysis of 1530 compounds [44]. Besides PAHs, significant levels of phthalates, pesticides, and industrial chemicals were reported. Levasseur et al. (2022) included OPFRs, brominated flame retardants, PCBs, pesticides, phthalates, and per- and polyfluoroalkyl substances [26]. In the latter study, TPP, EHDPP, TEHP, 4tBPDPP, B4IPPP, T4tBPP, TDMPP, and B4tBPPP median concentration were higher in SWBs worn on-duty than off-duty. EHDPP had the highest median concentration in SWBs worn on duty with no fire event (1273 ng/g). In the present study, the OPFR median concentration was 2–3 orders of magnitude lower than those reported by Levasseur et al. (2022). This may be due to the shorter deployment time of the SWBs in the present study (i.e., 20–90 min) compared to 6 days of on-duty deployment in the Levasseur et al. study. In this study, there were strong correlations between TPP and 2IPPDPP (r = 0.98, *p* < 0.001), TPP and TnBP (r = 0.88, *p* < 0.001), and TnBP and 2IPPDPP (r = 0.93, *p* < 0.001). Coincidentally, Phillips et al. (2017) [45] detected several isopropylated and tert-butylated triarylphosphate (ITP and TBPP) isomers in several commercial flame retardant mixtures including Firemaster (FM) 550 and 600, with mass fractions greater than 30% for ITPs (e.g., 2IPPDPP, 3IPPDPP, 4IPPDPP, 24DIPPDPP, B2IPPPP, B3IPPPP, B4IPPPP) in FM-550, and greater than 40% for TBPP (e.g., 4tBPDPP, B4tBPPP, and T4tBPP). TPP mass fractions were about 20% and 2% in FM-550 and FM-600, respectively.

### 4.3. Study Limitations

One crucial limitation of this study is the deployment time relative to absorption kinetics. O’Connell et al. (2022) reported that PAH concentrations of phenanthrene, pyrene, and fluorene remained in the linear uptake phase after 15 days [46]. Indeed, it may be possible that equilibrium was reached for naphthalene, acenaphthylene, and acenaphthene within the 30-day deployment time. For HMW PAHs, OPFRs, and PBDE, a linear uptake phase has been observed after 60 days [47]. However, the deployment of SWB in extreme conditions, such as high temperatures (up to 100 °C) and high turbulence, may affect the absorption kinetics, as discussed by Keir et al. (2023) [29]. Nevertheless, SWBs can still provide important information about firefighters’ potential exposure since combustion products, particularly in the gas phase, can be transported to the inside of the turnout gear through the interfaces between jackets–pants and hoods–jackets. These findings are crucial for understanding and improving occupational health and safety in firefighting, but it is important to note that the actual exposure levels may be higher or lower than what the SWBs indicate due to the limitations related to the deployment time and factors affecting the kinetics of absorption (e.g., temperature, air velocity). It is important to note that the cancer risk that can be attributed to the investigated carcinogenic PAHs (i.e., the B2 PAHs) will depend on the actual exposure level. As noted earlier, this study did not investigate actual exposure.

The second limitation of this study is the low rate of firefighters’ participation during fire suppression events. In this study, 52 participants enrolled in the study. Of those, only 24 participants were involved in fire suppression. However, only 18 firefighters remembered to place the SWBs outside the jacket, and not all the participants completed the questionnaire. By deploying more SWBs, it should be possible to increase the number of participants and the likelihood that those participants actually attend a fire suppression event. This could be performed in conjunction with a better recruitment and communication campaign aimed at increasing the participation rate.

Finally, by including other combustion products in the analysis of SWB, such as dioxins and furans, we can enhance the comprehensiveness of our findings, paving the way for more effective occupational health and safety measures in firefighting.

## 5. Conclusions

This study investigated the potential for firefighters to be occupationally exposed to PAHs and FRs in the fire station and during fire suppression. For this purpose, SWBs were used as passive samplers. The comprehensive sampling strategy employed, which included examination of different areas in four fire stations, fire truck cabins, and personal sampling of firefighters participating in fire suppression events, provided a robust dataset to understand the sources of PAHs and flame retardants in the firefighter occupational environment. The results showed that some areas in the fire station (i.e., VB) and truck cabins had higher levels of LMW PAH. Surprisingly, HMW PAHs in living room areas were as high as the vehicle bays and truck cabins. The source(s) of HMW PAH in the living room area remain unknown. Relative to fire stations, PAH levels in truck cabins were generally higher as they are likely transported from fire suppression events. Interestingly, TnBP, TCEP, and TCPP were ubiquitous in the fire stations, truck cabins, and office areas. Signature OPFRs were detected in the truck cabins; this is likely related to flammability standards established by the NHTSA.

Although it is challenging to precisely determine firefighters’ exposures to airborne PAHs and other chemicals encountered during fire suppression, the use of SWBs provides a reliable snapshot of exposure potential. Higher levels of PAHs were found in SWBs worn by firefighters who entered the fire. High PAHs and EHDPP were higher when heavy smoke was reported. This highlights the importance of the role of fire suppression in determining potential exposure. The gaps in the PPE interfaces allow the transport of chemicals from the surroundings to the inside of the turnout gear, increasing the exposure to the combustion by-products. This underscores the value and reliability of SWBs as passive samplers in assessing occupational exposure in firefighting environments.

Overall, the results obtained in this study provide important information regarding the sources of noteworthy toxicants in the firefighter occupational environment. While some analytes dominate the fire suppression environment (i.e., PAHs), other analytes are more abundant in station areas, truck cabins, and offices (i.e., OPFRs). The implications for firefighter exposure can guide future studies to more accurately define sources and actual exposure levels. More specifically, to better understand occupational sources of the chemicals examined herein and the associated risk of adverse health effects, future studies should (i) increase the number of firefighters participating in the study, (ii) incorporate environmental monitors alongside the deployed SWBs, (iii) effectively examine the areas surrounding the SWB deployment locations, and (iv) investigate the levels of FRs in materials contained in the fire station areas and truck cabins. An increase in firefighter participation will permit more effective investigations regarding the impact of fire type, fire duration, and PPE use on SWB analyte concentrations. Future work should also employ biomonitoring methods to quantify firefighter exposures to the investigated analytes; the results obtained in this study can guide the design of those follow-up studies.

## Data Availability

The raw data supporting the conclusions of this article will be made available by the authors upon request.

## Supplementary Materials

The following supporting information can be downloaded at https://www.mdpi.com/article/10.3390/toxics12090677/s1, Table S1: List of target analytes, CAS numbers, abbreviations, and suppliers; Table S2: GC-MS MRM transitions with corresponding internal standards; Table S3: Method detection limits (MDL) and recovery experiment results; Table S4: Descriptive information about firefighting events; Table S5: Correlation matrix of analytes detected in station area SWB Samples; Table S6: Correlation matrix of analytes detected in firefighter SWB Samples; Figure S1: Comparison of detection frequency (%) in fire station (n = 12) and office areas (n = 10); Figure S2: Box plots summarizing concentrations (ng/g) of (a) BDE-47, (b) TDCPP, and (c) TCPP in the fire station areas; Figure S3: Box plot summarizing concentrations (ng/g) of EHDPP in silicone wristbands donned outside the jacket of firefighters responding to a fire.
