# Supplementary material for: Occupational Exposure of On-Shift Ottawa Firefighters to Flame Retardants and Polycyclic Aromatic Hydrocarbons
Source: Toxics. 2024 Sep 17;12(9):677. doi: 10.3390/toxics12090677 (PMC11435908; doi:10.3390/toxics12090677)
Supplement: Supplementary file 1 [file toxics-12-00677-s001.zip › toxics-3147777-supplementary.pdf]

Supplementary Information

Occupational Exposure of On-shift Ottawa Firefighters to Flame Retardants and Polycyclic Aromatic Hydrocarbons

William Papas[1§], Rocio Aranda-Rodriguez[1§*],Cariton Kubwabo[1], Xinghua Fan[1], Janet S. L. Lee[1], Emma M. L. Fantin[1], Elita D. Zheng[1], Jennifer L. A. Keir[2], Dave Matschke[3], Jules M. Blais[2], Paul A. White[1*]

[1]Environmental Health Science and Research Bureau, Health Canada. 251 Sir Frederick Banting Driveway, Ottawa, Ontario K1A 0K9, Canada

[2]Department of Biology, University of Ottawa, 30 Marie Curie, Ottawa, Ontario, K1N 6N5, Canada.

[3]Ottawa Fire Services, 1445 Carling Ave., Ottawa, Ontario, K1Z 7L9, Canada.

[§] Contributed equally
* Correspondence: Rocio.aranda-rodriguez@hc-sc.gc.ca; paul.white@hc-sc.gc.ca

Table of Contents

Chemical Analyses.. 3

Table S1. List of target analytes, CAS numbers, abbreviations, and suppliers 4

Table S2. GC-MS MRM transitions with corresponding internal standards................................ 7

Table S3. Method detection limits (MDL) and recovery experiment results 11

Table S4. Descriptive information about firefighting events…... 14

Table S5. Correlation matrix of analytes detected in station area SWB Samples.. 16

Table S6. Correlation matrix of analytes detected in firefighter SWB Samples.. 17

Figure S1. Comparison of detection frequency (%) in fire station and office areas.................... 18

Figure S2. Box-plots summarizing concentrations (ng/g) of (a) BDE-47, (b) TDCPP, and (c) TCPP in fire station areas. 19

Figure S3. Box-plot summarizing concentrations (ng/g) of EHDPP in silicone wristbands donned outside the jacket of firefighters responding to a fire. 20

Chemical Analyses

PAHs and PBDEs

Sample extracts were analyzed using a Trace GC Ultra gas chromatograph (GC) coupled with a TSQ 8000 triple quadrupole mass spectrometer (MS/MS) operated in multiple reaction (MRM) mode. The GC system was equipped with a split/splitless injector operated in splitless mode with 0.8 min of splitless time, a split flow of 30 mL/min, and a constant temperature of 300 °C. The GC was fitted with a ZB-Semivolatiles GC column (30 m x 0.25 mm x 0.25 µm) from Phenomenex (Torrace, CA, USA). The carrier gas was helium set at a constant flow rate of 1 mL/min. The oven temperature was initially held at 55°C for 2 min, then ramped at 18°C/min to 320°C and held for 1 minute at final temperature. The source temperature was 300°C and transfer line temperature was 280°C. The MS triple quadrupole mass analyzer was used with Q1 and Q3 peak width set at 0.7 (FWHM) and a cycle time of 0.2 seconds.

OPFRs

Sample extracts were analyzed using an Agilent 7890N gas chromatographer (GC) coupled with Waters Xevo TQ-GC mass spectrometer (MS/MS), operated inmultiple reaction (MRM mode. The ion source and GC interface temperatures were set at 230 °C and 280 °C, respectively. The GC column was a Zebron ZB-5MS (30m× 0.25 mm x 0.25 µm) from Phenomenex (Torrance, CA, USA). The carrier gas was helium with a constant flow of 1 mL/min. The oven temperature was initially held at 50 °C for 1.2 min, ramped to 250 °C at 10 °C/min, ramped to 260 °C at 1 °C /min, then ramped to 300 °C at 25 °C /min, and finally held at 300 °C for 14 min. The GC injector was equipped with a programmable-temperature vaporizer inlet (PTV) which was operated in solvent vent mode. The initial inlet temperature was held at 70 °C for 0.04 min, ramped to 300 °C at 700 °C/min, and held at 300 °C thereafter until the end of the GC/MS analysis. Vent pressure was set at 10 kPa with vent flow of 75 mL /min ending at 0.02 min. Purge flow was 50 ml /min after 1.0 min. The injection volume was 2 µL.

Table S1. List of target analytes, CAS numbers, abbreviations, and suppliers

Analyte	CAS Number	Abbreviation	Supplier
PAH			
Low Molecular Weight (LMW) PAH			
Naphthalene[3]	91-20-3	Naph	Wellington Laboratories
Acenaphthylene	208-96-8	Acethy	Wellington Laboratories
Acenaphthene	83-32-9	Acenap	Wellington Laboratories
Fluorene	86-73-7	Fluo	Wellington Laboratories
Phenanthrene	85-01-8	Phen	Wellington Laboratories
Anthracene	120-12-7	Anth	Wellington Laboratories
Hight Molecular Weight (HMW) PAH			
Fluoranthene	206-44-0	Fluor	Wellington Laboratories
Pyrene	129-00-0	Pyr	Wellington Laboratories
Benz[*a*]anthracene[1]	56-55-3	BaA	Wellington Laboratories
Chrysene[3]	218-01-9	Chry	Wellington Laboratories
Benzo[*b*]fluoranthene[3]	205-99-2	BbF	Wellington Laboratories
Benzo[*k*]fluoranthene[3]	207-08-9	BkF	Wellington Laboratories
Benzo[*a*]pyrene[1]	50-32-8	BaP	Wellington Laboratories
Indeno[*1, 2, 3-cd]*pyrene	193-39-5	Inde	Wellington Laboratories
Dibenz[*a, h]*anthracene[2]	53-70-3	DBahA	Wellington Laboratories
Benzo[*g, h, i*]perylene	191-24-2	Benzoghi	Wellington Laboratories
PBDEs			
BDE-28	41318-75-6	BDE-28	Wellington Laboratories
BDE-47	5436-43-1	BDE-47	Wellington Laboratories
BDE-66	189084-61-5	BDE-66	Wellington Laboratories

BDE-85	182346-21-0	BDE-85	Wellington Laboratories
BDE-99	60348-60-9	BDE-99	Wellington Laboratories
BDE-100	189084-64-8	BDE-100	Wellington Laboratories
BDE-153	68631-49-2	BDE-153	Wellington Laboratories
BDE-154	207122-15-4	BDE-154	Wellington Laboratories
BDE-183	207122-16-5	BDE-183	Wellington Laboratories
OPFRs			
Dibutyl phenyl phosphate	2528-36-1	DBPP	AccuStandard
Isopropyl diphenyl phosphate	60763-39-5	IPDPP	AccuStandard
Butyl diphenyl phosphate	2752-95-6	BDPP	AccuStandard
2-Ethylhexyl diphenyl phosphate	1241-94-7	EHDPP	AccuStandard
Triphenyl phosphate	115-86-6	TPP	AccuStandard
2-Isopropylphenyl diphenyl phosphate	64532-94-1	2IPPDPP	Wellington Laboratories
3-Isopropylphenyl diphenyl phosphate	69515-46-4	3IPPDPP	Wellington Laboratories
4-Isopropylphenyl diphenyl phosphate	55864-04-5	4IPPDPP	Wellington Laboratories
2-tert-Butylphenyl diphenyl phosphate	N/A	2tBPDPP	Wellington Laboratories
3-tert-Butylphenyl diphenyl phosphate	N/A	3tBPDPP	Wellington Laboratories
4-tert-Butylphenyl diphenyl phosphate	981-40-8	4tBPDPP	Wellington Laboratories
2,4-Diisopropylphenyl diphenyl phosphate	96107-55-0	24DIPPDPP	Wellington Laboratories
Bis(2-isopropylphenyl) phenyl phosphate	69500-29-4	B2IPPPP	Wellington Laboratories
Bis(3-isopropylphenol) phenyl phosphate	69500-30-7	B3IPPPP	Wellington Laboratories
Bis(4-isopropylphenyl) phenyl phosphate	55864-07-8	B4IPPPP	Wellington Laboratories
Bis(2-tert-butylphenyl) phenyl phosphate	65652-41-7	B2tBPPP	Wellington Laboratories
Bis(3-tert-butylphenyl) phenyl phosphate	N/A	B3tBPPP	Wellington Laboratories

Bis(4-tert-butylphenyl) phenyl phosphate	115-87-7	B4tBPPP	Wellington Laboratories
Bis(2,4-diisopropylphenyl) phenyl phosphate	N/A	B24DIPPPP	Wellington Laboratories
Tri-o-cresyl phosphate	78-30-8	TOTP	AccuStandard
Tri-m-cresyl phosphate	563-04-2	TMTP	AccuStandard
Tri-p-cresyl phosphate	78-32-0	TPTP	AccuStandard
Tris(3,5-dimethylphenyl) phosphate	25653-16-1	T35DMPP	Wellington Laboratories
Tris(3,4-dimethylphenyl) phosphate	3862-11-1	T34DMPP	Wellington Laboratories
Tris(2-isopropylphenyl) phosphate	64532-95-2	T2IPPP	Wellington Laboratories
Tris(3-isopropylphenyl) phosphate	72668-27-0	T3IPPP	Wellington Laboratories
Tris(4-isopropylphenyl) phosphate	2502-15-0	T4IPPP	Wellington Laboratories
Tris(3-tert-butylphenyl) phosphate	N/A	T3tBPP	Wellington Laboratories
Tris(4-tert-butylphenyl) phosphate	78-33-1	T4tBPP	Wellington Laboratories
Tri-n-butyl phosphate	126-73-8	TnBP	AccuStandard
Tripentyl phosphate	2528-38-3	TPeP	AccuStandard
Tri (2-butoxyethyl) phosphate	78-51-3	TBEP	AccuStandard
Tris(2-ethylhexyl) phosphate	78-42-2	TEHP	AccuStandard
Tris(2-chloroethyl)phosphate	115-96-8	TCEP	AccuStandard
Tris(1-chloro-2-propyl) phosphate	13674-84-5	TCPP	AccuStandard
Tris(1,3-dichloro-2-propyl)phosphate	13674-87-8	TDCPP	AccuStandard
Tris(2-chloroethyl)phosphate-d_{12}	Internal standard	D-TCEP	CDN Istopes Laboratories
Triphenyl Phosphate-$^{13}C_{18}$	Internal standard	M-TPP	Wellington Laboratories

[1]IARC Group 1, carcinogenic to humans; [2]IARC Group 2A, probably carcinogenic to humans; [3]IARC Group 2B, possibly carcinogenic to humans

Table S2. GC-MS MRM transitions with corresponding internal standards

	MRM-Q[1]			MRM-C[2]			Internal Standard			
Analyte	Precursor	Product	CE	Precursor	Product	CE	Corresponding Internal Standard	Precursor	Product	CE
**PAHs**										
Naphthalene	128.1	102	15				Naphthalene-d_8	136.1	108.1	20
Acenaphthylene	152.1	126.1	25	152.1	151.1	15	Acenaphthylene-d_8	160.1	158.1	15
Acenaphthene	153.1	127.1	25	153.1	152.1	15				
Fluorene	166.1	165.1	15							
Phenanthrene	178.1	152.2	20				Phenanthrene-d_{10}	188.2	160.1	20
Anthracene	178.1	152.1	20							
Fluoranthene	202.1	200.1	30				Fluoranthene-d_{10}	212.2	208.1	35
Pyrene	202.1	200.1	35							
p-Terphenyl-d_{14}	244.2	240.2	25							
Benz[*a*]anthracene	228.1	226.2	30				Benz[*a*]anthracene-d_{12}	240.2	236.2	30
Chrysene	228.1	226.1	30				Chrysene-d_{12}	240.2	236.1	30
Benzo[*b*]fluoranthene	252.1	250.1	30				Benzo[*k*]fluoranthene-d_{12}	264.2	260.2	30
Benzo[*k*]fluoranthene	252.1	250.1	30				Benzo[*k*]fluoranthene-d_{12}	264.2	260.1	35
Benzo[*a*]pyrene	252.1	250.1	35				Benzo[a]pyrene-d_{12}	264.2	260.2	40
Indeno[*1,2,3-cd*]pyrene	276.1	274.1	35				Indeno[*1,2,3-cd]*pyrene-d_{12}	288.2	284.2	40
Dibenz[*a, h*]anthracene	278.1	276.1	30				Dibenz[*a, h*]anthracene-d_{14}	292.2	288.2	35
Benzo[*g,h,i*]perylene	276.1	274.1	40				Benzo[*g,h,i*]perylene-d_{12}			
**PBDEs**										
BDE-28	246	139.1	26	407.8	248	18	BDE-28-IS	258.1	150.1	28

BDE-47	323.9	217	28	485.8	325.9	20	BDE-47-IS	497.8	337.9	18
BDE-66	323.9	216.9	28	458.8	325.9	20				
BDE-85	403.8	296.9	28	563.7	403.7	20	BDE-154-IS	495.7	495.7	10
BDE-99	403.8	297	32	565.7	405.7	18	BDE-99-IS	415.9	308	30
BDE-100	403.8	296.9	30	563.7	403.7	18	BDE-100-IS	415.8	307.9	30
BDE-153	483.8	323.9	38	643.7	483.7	14	BDE-153-IS	495.7	495.7	10
BDE-154	643.6	483.8	12	483.8	402.8	18	BDE-154-IS	495.7	495.7	10
BDE-183	561.7	454.7	38				BDE-183-IS	573.6	573.6	10
**OPFRs**										
Dibutyl phenyl phosphate	286	175	21	286	231	6	Triphenyl phosphate-$^{13}C_{18}$	344	343	6
Isopropyl diphenyl phosphate	292	170	15	292	250	6	Triphenyl phosphate-$^{13}C_{18}$	344	343	6
Butyl diphenyl phosphate	306	170	19	306	250	8	Triphenyl phosphate-$^{13}C_{18}$	344	343	6
2-Ethylhexyl diphenyl phosphate	251	77	13	251	153	8	Triphenyl phosphate-$^{13}C_{18}$	344	343	6
Triphenyl phosphate	326	233	22	326	325	22	Triphenyl phosphate-$^{13}C_{18}$	344	343	6
2-Isopropylphenyl diphenyl phosphate	368	118	14	368	251	18	Triphenyl phosphate-$^{13}C_{18}$	344	343	6
3-Isopropylphenyl diphenyl phosphate	368	118	18	368	251	14	Triphenyl phosphate-$^{13}C_{18}$	344	343	6
4-Isopropylphenyl diphenyl phosphate	368	118	18	368	353	14	Triphenyl phosphate-$^{13}C_{18}$	344	343	6
2-tert-Butylphenyl diphenyl phosphate	367	178	24	382	367	14	Triphenyl phosphate-$^{13}C_{18}$	344	343	6
3-tert-Butylphenyl diphenyl phosphate	367	178	24	382	367	14	Triphenyl phosphate-$^{13}C_{18}$	344	343	6
4-tert-Butylphenyl diphenyl phosphate	367	178	26	382	367	14	Triphenyl phosphate-$^{13}C_{18}$	344	343	6
2,4-Diisopropylphenyl diphenyl phosphate	410	145	26	410	160	14	Triphenyl phosphate-$^{13}C_{18}$	344	343	6

Bis(2-isopropylphenyl) phenyl phosphate	410	118	18	410	251	24	Triphenyl phosphate-$^{13}C_{18}$	344	343	6
Bis(3-isopropylphenol) phenyl phosphate	410	118	18	410	251	24	Triphenyl phosphate-$^{13}C_{18}$	344	343	6
Bis(4-isopropylphenyl) phenyl phosphate	410	251	26	410	395	18	Triphenyl phosphate-$^{13}C_{18}$	344	343	6
Bis(2-tert-butylphenyl) phenyl phosphate	423	367	16	438	423	14	Triphenyl phosphate-$^{13}C_{18}$	344	343	6
Bis(3-tert-butylphenyl) phenyl phosphate	423	367	18	438	367	24	Triphenyl phosphate-$^{13}C_{18}$	344	343	6
Bis(4-tert-butylphenyl) phenyl phosphate	423	367	18	438	423	16	Triphenyl phosphate-$^{13}C_{18}$	344	343	6
Bis(2,4-diisopropylphenyl) phenyl phosphate	494	145	32	494	160	16	Triphenyl phosphate-$^{13}C_{18}$	344	343	6
Tri-o-cresyl phosphate	368	277	11	368	367	7	Triphenyl phosphate-$^{13}C_{18}$	344	343	6
Tri-m-cresyl phosphate	368	261	13	368	367	7	Triphenyl phosphate-$^{13}C_{18}$	344	343	6
Tri-p-cresyl phosphate	368	261	13	368	367	9	Triphenyl phosphate-$^{13}C_{18}$	344	343	6
Tris(3,5-dimethylphenyl) phosphate	410	193	24	410	395	16	Triphenyl phosphate-$^{13}C_{18}$	344	343	6
Tris(3,4-dimethylphenyl) phosphate	410	193	26	410	288	18	Triphenyl phosphate-$^{13}C_{18}$	344	343	6

Tris(2-isopropylphenyl) phosphate	452	118	18	452	335	14	Triphenyl phosphate-$^{13}C_{18}$	344	343	6
Tris(3-isopropylphenyl) phosphate	452	118	22	452	335	14	Triphenyl phosphate-$^{13}C_{18}$	344	343	6
Tris(4-isopropylphenyl) phosphate	437	395	18	452	437	18	Triphenyl phosphate-$^{13}C_{18}$	344	343	6
Tris(3-tert-butylphenyl) phosphate	479	367	22	479	423	14	Triphenyl phosphate-$^{13}C_{18}$	344	343	6
Tris(4-tert-butylphenyl) phosphate	494	367	34	494	479	16	Triphenyl phosphate-$^{13}C_{18}$	344	343	6
Tri-n-butyl phosphate	211	99	10	211	155	7	Tris(2-chloroethyl) phosphate-d_{12}	261	131	16
Tripentyl phosphate	239	99	10	239	169	7	Tris(2-chloroethyl) phosphate-d12	261	131	16
Tri (2-butoxyethyl) phosphate	299	57	12	299	199	7	Tris(2-chloroethyl) phosphate-d_{12}	261	131	16
Tris(2-ethylhexyl) phosphate	99	81	19	113	57	17	Tris(2-chloroethyl) phosphate-d_{12}	261	131	16
Tris(2-chloroethyl)phosphate	249	125	14	249	187	10	Tris(2-chloroethyl) phosphate-d_{12}	261	131	16
Tris(1-chloro-2-propyl) phosphate	277	125	11	277	201	7	Tris(2-chloroethyl) phosphate-d_{12}	261	131	16
Tris(1,3-dichloro-2-propyl)phosphate	381	159	13	381	271	9	Tris(2-chloroethyl) phosphate-d_{12}	261	131	16

[1]MRM quantitation ion; [2]MRM confirmation ion

Table S3. Method detection limits (MDL) and recovery experiment results

Analyte	MDL (ng/g SWB)	Recovery[1] (%)	RSD[1] (%)
**PAHs**			
**LMW PAHs**			
Naphthalene	0.791	147	1
Acenaphthylene	0.189	93	3
Acenaphthene	1.030	93	6
Fluorene	1.502	103	6
Phenanthrene	1.287	95	3
Anthracene	0.465	89	3
**HMW PAHs**			
Fluoranthene	0.578	86	1
Pyrene	1.137	88	1
Benz[*a*]anthracene	0.700	103	38
Chrysene	1.637	119	16
Benzo[*b*]fluoranthene	0.316	75	6
Benzo[*k*]fluoranthene	0.514	89	4
Benzo[*a*]pyrene	0.462	78	5
Indeno[*1, 2, 3-cd]*pyrene	0.563	99	17
Dibenz[*a, h*]anthracene	0.675	111	8
Benzo[*g,h,i*]perylene	0.427	82	5
**PBDEs**			
BDE-28	0.554	115	4
BDE-47	0.433	125	4
BDE-66	0.443	138	6
BDE-85	0.677	102	6
BDE-99	0.604	105	5
BDE-100	0.573	230	28
BDE-153	0.413	129	42
BDE-154	0.767	108	35
BDE-183	0.722	106	7
**OPFRs**			
Dibutyl phenyl phosphate	3.715	77	9
Isopropyl diphenyl phosphate	2.997	99	4
Butyl diphenyl phosphate	1.761	117	2
2-Ethylhexyl diphenyl phosphate	1.708	88	2
Triphenyl phosphate	5.329	98	2

2-Isopropylphenyl diphenyl phosphate	2.850	103	2
3-Isopropylphenyl diphenyl phosphate	2.683	91	3
4-Isopropylphenyl diphenyl phosphate	4.975	107	6
2-tert-Butylphenyl diphenyl phosphate	2.337	97	2
3-tert-Butylphenyl diphenyl phosphate	4.089	82	1
4-tert-Butylphenyl diphenyl phosphate	8.301	83	2
2,4-Diisopropylphenyl diphenyl phosphate	1.345	110	6
Bis(2-isopropylphenyl) phenyl phosphate	2.644	85	3
Bis(3-isopropylphenol) phenyl phosphate	1.375	83	4
Bis(4-isopropylphenyl) phenyl phosphate	3.885	71	12
Bis(2-tert-butylphenyl) phenyl phosphate	4.298	103	7
Bis(3-tert-butylphenyl) phenyl phosphate	3.920	73	7
Bis(4-tert-butylphenyl) phenyl phosphate	8.567	85	20
Bis(2,4-diisopropylphenyl) phenyl phosphate	8.671	84	14
Tri-o-cresyl phosphate	5.088	83	7
Tri-m-cresyl phosphate	3.317	88	6
Tri-p-cresyl phosphate	5.623	79	3
Tris(3,5-dimethylphenyl) phosphate	4.804	74	9

Tris(3,4-dimethylphenyl) phosphate	7.223	79	20
Tris(2-isopropylphenyl) phosphate	3.065	69	5
Tris(3-isopropylphenyl) phosphate	9.756	85	13
Tris(4-isopropylphenyl) phosphate	11.853	94	1
Tris(3-tert-butylphenyl) phosphate	11.592	85	4
Tris(4-tert-butylphenyl) phosphate	8.240	68	9
Tri-n-butyl phosphate	1.459	111	9
Tripentyl phosphate	1.838	77	10
Tri (2-butoxyethyl) phosphate	3.795	69	10
Tris(2-ethylhexyl) phosphate	3.339	98	4
Tris(2-chloroethyl)phosphate	1.870	99	3
Tris(1-chloro-2-propyl) phosphate	7.128	73	5
Tris(1,3-dichloro-2-propyl)phosphate	3.227	104	9

[1]Results from recovery experiments testing the sonication extraction method. PAHs spiked at 18.75 ng. PBDEs spiked at 7.5 ng. OPFRs spiked at 75 ng.

Table S4. Descriptive information about firefighting events. PAH and OPFR concentrations (ng/g) were measured in SWBs donned outside the turnout jacket.

ID	Enter Fire	Fire type	Material	Stage of Fire	Peak intensity	Smoke	Smoke color	LMW PAH	HMW PAH	Total PAHs	Aryl OPFRs	Alkyl OPFRs	Halogenated OPFRs	Total OPFRs
FF1	YES	Residential	Pot on stove Furniture, wall, ceiling, garbage	Decay	Smoldering/smoke with no visible flames	M	LG	1040	32	1072	22	138	310	470
FF2	YES	Residential	Furniture, carpets, wall, ceiling	Fully developed	Heavy smoke with flames pushing from the structure/object	VH	DG_BB_DB	7389	168	7557	128	889	101	1588
FF3	YES	Residential	Furniture, clothes	Oxygen/ventilation limited	Smoke with flames	VH	BB	2254	19	2272	67	14	0.1	81
FF4	YES	Commercial	Walls, ceilings	Decay	Smoldering/smoke with no visible flames	VH	DG	5203	98	5301	31	5.6	100	137
FF5	YES	Industrial	Furniture, gas fuel	Growing	Smoke with flames	VH	W_DG_BB	1135	23	1159	31	555	55	641
FF6	YES	Residential	Furniture, carpets, walls, ceiling, clothes, electronics, garbage	Decay	Heavy smoke with flames pushing from the structure/object	L	LG_DG-DB	758	14	772	87	223	87	397
FF7	YES	Automotive	Delivery vehicle, mix of goods (clothing, electronics)	Decay	Heavy smoke with flames pushing from the structure/object	M	DG_BB_DB	427	2.8	430	8.2	8.1	10	26
FF8	YES	Residential	Walls, ceiling, auto	Decay	Heavy smoke with flames pushing from the structure/object	VH	DG_DB	403	26	429	386	595	74	1055
FF9	YES	Residential	Furniture	Decay	Smoldering/smoke with no visible flames	VH	DG	247	1.2	248	9.1	24	0.3	33
FF10	YES	Residential	Clothes, garbage	Oxygen/ventilation limited	Smoldering/smoke with no visible flames	L	LG	41	2.7	44	185	520	7.7	713
FF11	YES	Residential	Electronics, clothes	Growing	Smoke with minimal flames	L	W-LG	15	2.2	17	6.6	5.4	3.8	16

FF12	NO	Residential	Walls, ceiling, auto tyres	Did not enter the structure	Smoke with minimal flames	L	LG	19	2.3	22	8.1	95	0.6	103
FF13	NO	Residential	Walls, ceiling	Did not enter the structure	Smoke with minimal flames	M	LG	48	0.6	49	4.6	8.6	0.9	14
FF14	NO	Residential	Electronics, deck	Did not enter the structure	Smoldering/smoke with no visible flames	M	LG	33	7.5	41	3.9	3.6	1.4	9
FF15	NO	Residential	Furniture, carpet, walls, ceiling, clothes, garbage	Did not enter the structure	Heavy smoke with flames pushing from the structure/object	L	LG	130	3.3	133	269	53	16	338
FF16	NO	Residential	Furniture, walls, ceiling, garbage, auto tyres	Did not enter the structure	Heavy smoke with flames pushing from the structure/object	L	DB	25	4.3	29	11	50	6	67
FF17	NO	Residential	Furniture, carpet, walls, ceiling	Did not enter the structure	Smoke with minimal flames	M	W-LG_DG	11	5.1	17	12	26	8	46
FF18	NO	Residential	Pot on stove	Incipient	Smoldering/smoke with no visible flames	M	LG	9	2.0	11	26	561	2	590

FF: Firefighter. Smoke: L: Light haze/smoke M: medium haze/smoke, VH: very heavy smoke; H: heavy haze/smoke. Smoke colour: LG: light gray; W_LG: white light gray; DG: dark gray, W-LB: white-light brown; BB: black brown; DB: dark brown.

Table S5. Correlation matrix of analytes detected in station area SWB Samples. Values shown are Pearson correlation coefficients. Coefficients that are not significant at p<0.05 are not displayed. *p<0.05, **p<0.01, ***p<0.001. Full names of analytes are provided in Table S1.

	Naph	Acethy	Acenap	Fluo	Phen	Anth	Fluor	Pyr	BaA	Inde	DBahA	Benzoghi	BDE-47	EHDPP	TPP	2IPPDPP	TnBP	TCEP	TCPP	TDCPP
**Naph**	1	0.58***	0.77***	0.82***	0.82***	0.66***	0.62***	0.43***					-0.33**						0.36**	
**Acethy**		1	0.47***	0.57***	0.56***	0.37**	0.82***	0.75***					-0.42***	0.27*						
**Acenap**			1	0.92***	0.69***	0.58***	0.46***			0.30*		0.25*	-0.36**	0.50***	0.46***			-0.27*	0.37**	0.47***
**Fluo**				1	0.85***	0.6***	0.53***	0.24*		0.36**			-0.36**	0.42***	0.34**					0.32**
**Phen**					1	0.83***	0.72***	0.46***					-0.26*	0.31**					0.28*	
**Anth**						1	0.58***	0.32**						0.30*					0.37**	
**Fluor**							1	0.92***	0.35**				-0.30*	0.26*						
**Pyr**								1	0.49***				-0.29*							
**BaA**									1	-0.43***	-0.43***								0.24*	
**Inde**										1	0.79***	0.55***	-0.29*				0.46***	-0.23*		
**DBahA**											1	0.56***	-0.27*				0.56***	-0.27*		
**Benzoghi**												1	-0.23*					-0.26*		
**BDE-47**													1					0.60***		
**EHDPP**														1	0.67***					0.68***
**TPP**															1	0.28*				0.73***
**2IPPDPP**																1				0.53***
**TnBP**																	1			
**TCEP**																		1		
**TCPP**																			1	
**TDCPP**																				1

Table S6. Correlation matrix of analytes detected in firefighter SWB Samples. Values shown are Pearson correlation coefficients. Coefficients that are not significant at p<0.05 are not displayed.*p<0.05, **p<0.01, ***p<0.001. Full names of analytes are provided in Table S1.

	Naph	Acethy	Acenap	Fluo	Phen	Anth	Fluor	Pyr	BaA	Chry	BbF	BaP	Benzoghi	EHDPP	TPP	2IPPDPP	TnBP	TBEP	TDCPP
**Naph**	1	0.86***	0.78***	0.82***	0.89***	0.79***	0.93***	0.88***	0.90***	0.96***	0.97***	0.94***	0.97***						0.72***
**Acethy**		1	0.99***	0.99***	0.99***	0.99***	0.97***	0.98***	0.95***	0.76***	0.88***	0.85***	0.87***					0.55*	
**Acenap**			1	0.99***	0.97***	0.99***	0.93***	0.96***	0.92***	0.66**	0.80***	0.78***	0.80***					0.59*	
**Fluo**				1	0.99***	0.99***	0.95***	0.98***	0.94***	0.72***	0.85***	0.82***	0.84***					0.56*	
**Phen**					1	0.98***	0.99***	0.99***	0.97***	0.80***	0.91***	0.89***	0.90***					0.54*	
**Anth**						1	0.94***	0.98***	0.94***	0.69**	0.82***	0.81***	0.82***					0.60**	
**Fluor**							1	0.99***	0.98***	0.87***	0.95***	0.94***	0.95***					0.51*	0.57*
**Pyr**								1	0.98***	0.82***	0.91***	0.90***	0.91***					0.56*	0.48*
**BaA**									1	0.86***	0.91***	0.92***	0.91***					0.60**	0.55*
**Chry**										1	0.92***	0.95***	0.95***						0.81***
**BbF**											1	0.93***	0.98***						0.73***
**BaP**												1	0.95***						0.72***
**Benzoghi**													1						0.69**
**EHDPP**														1				0.58*	
**TPP**															1	0.98***	0.88***		
**2IPPDPP**																1	0.93***		
**TnBP**																	1		
**TBEP**																		1	
**TDCPP**																			1

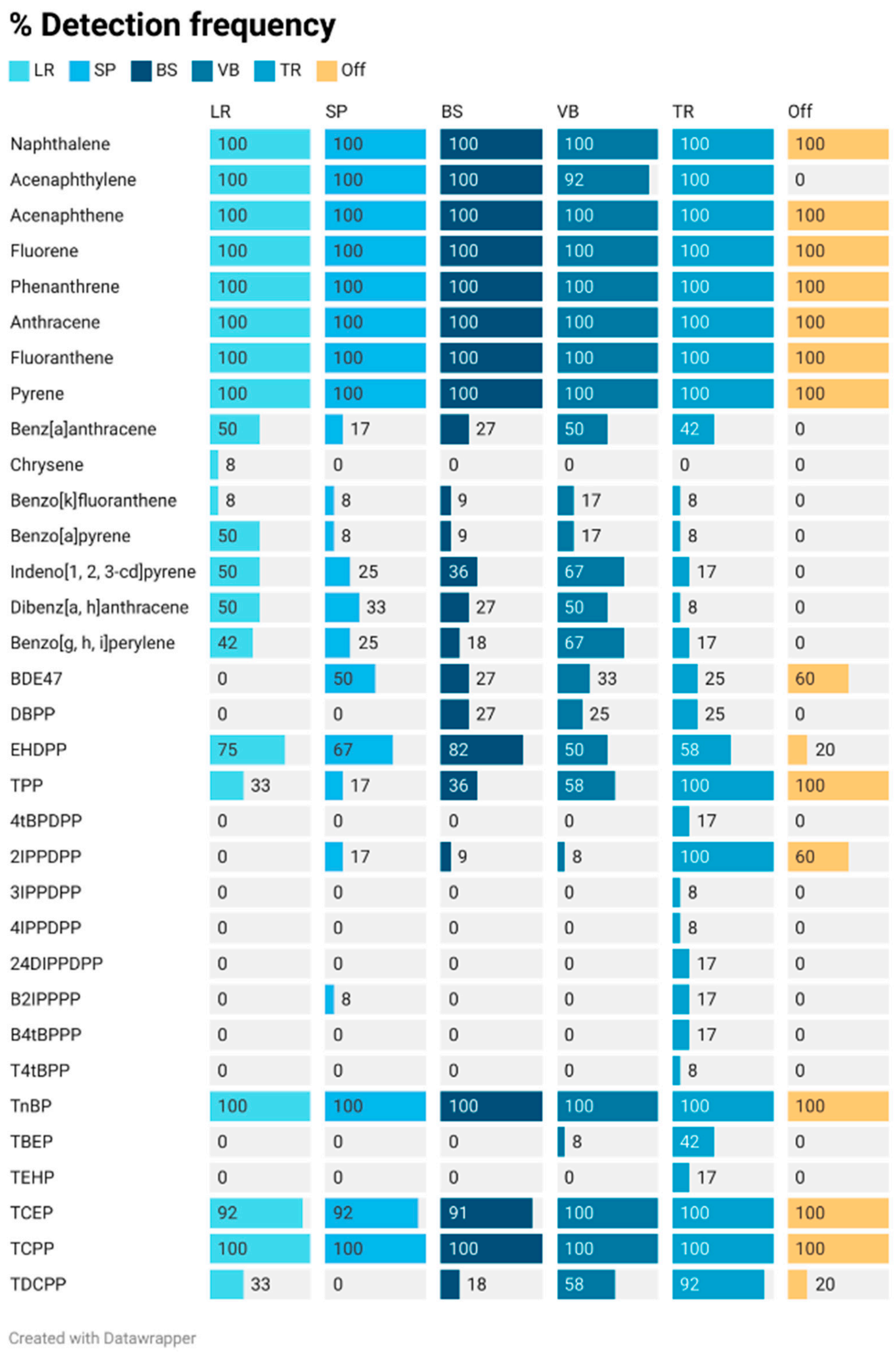

Figure S1. Comparison of detection frequency (%) in fire station (n=12) and office areas (n=10). Analytes that were not detected are not shown.

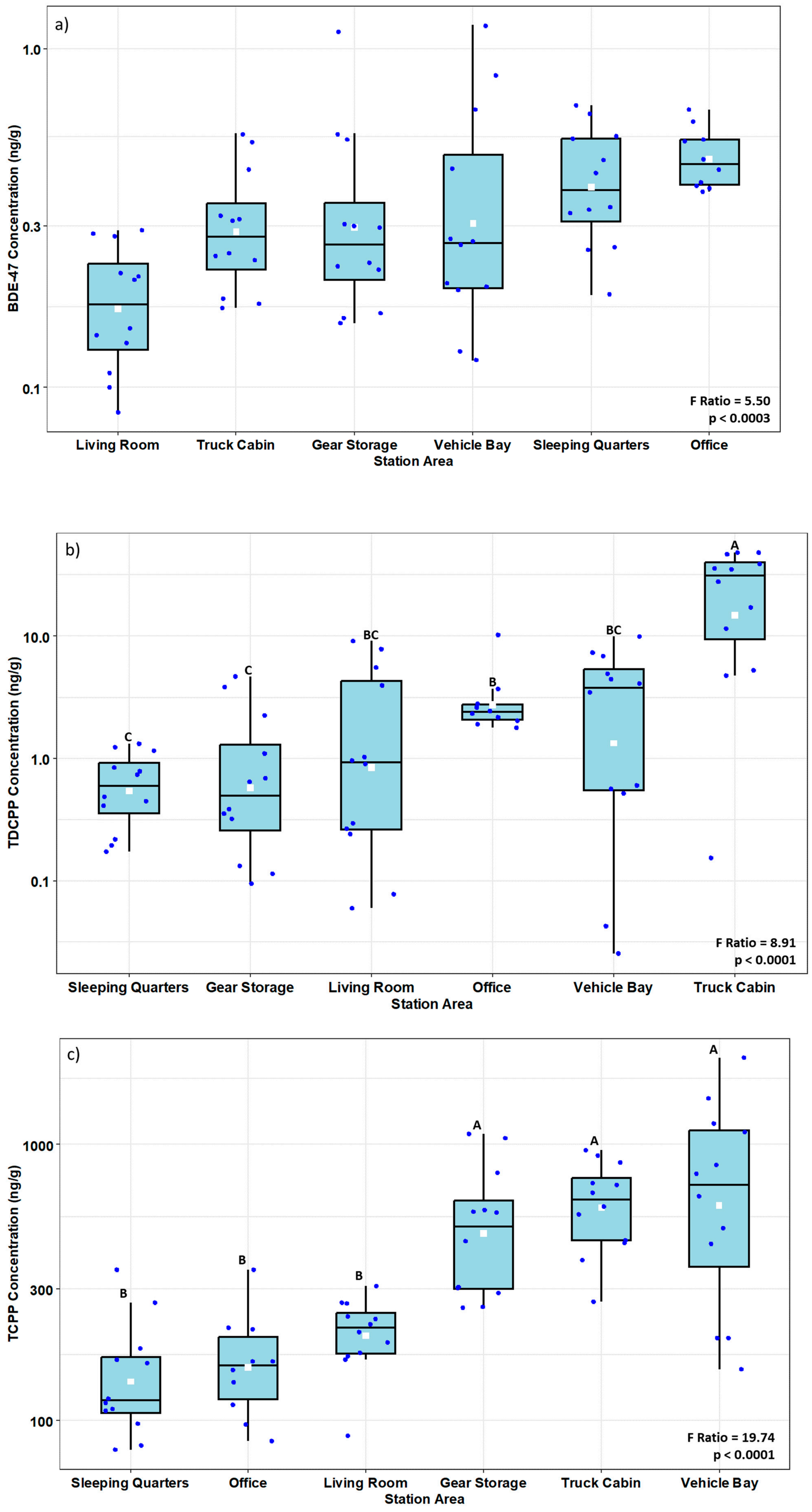

Figure S2. Box-plots summarizing concentrations (ng/g) of (a) BDE-47, (b) TDCPP, and (c) TCPP in fire station areas. Box limits represent the interquartile range (i.e., 25th to 75th percentile), the white squares represent the geometric mean values, the blue dots indicate the values for each observation, solid line represents the group median, and the whiskers extend to the 5th and 95th percentiles. The results of ANOVAs for station area effects are shown on the bottom right.

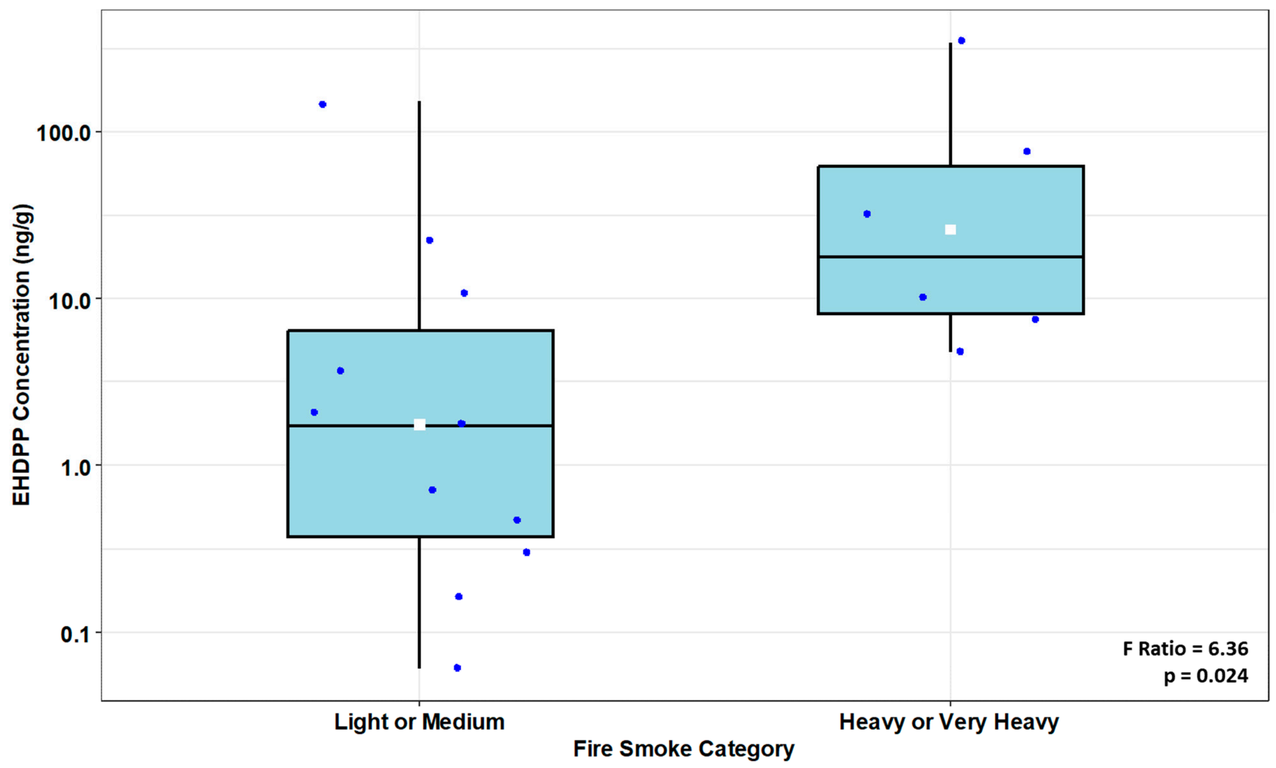

Figure S3. Box-plot summarizing concentrations (ng/g) of EHDPP in silicone wristbands donned outside the jacket of firefighters responding to a fire. Box limits represent the interquartile range (i.e. 25th to 75th percentile), the white squares represent the geometric mean values, the blue dots indicate the values for each observation, the solid line represents the group median, and the whiskers extend to the 5th and 95th percentiles. The result of an ANOVA for a smoke level effect is shown on the bottom right.
